# The Sense of Confidence during Probabilistic Learning: A Normative Account

**DOI:** 10.1371/journal.pcbi.1004305

**Published:** 2015-06-15

**Authors:** Florent Meyniel, Daniel Schlunegger, Stanislas Dehaene

**Affiliations:** 1 Cognitive Neuroimaging Unit, U992, INSERM, Gif/Yvette, France; 2 NeuroSpin Center, DSV/I2BM, CEA, Gif/Yvette, France; 3 Collège de France, Paris, France; Oxford University, UNITED KINGDOM

## Abstract

Learning in a stochastic environment consists of estimating a model from a limited amount of noisy data, and is therefore inherently uncertain. However, many classical models reduce the learning process to the updating of parameter estimates and neglect the fact that learning is also frequently accompanied by a variable “feeling of knowing” or confidence. The characteristics and the origin of these subjective confidence estimates thus remain largely unknown. Here we investigate whether, during learning, humans not only infer a model of their environment, but also derive an accurate sense of confidence from their inferences. In our experiment, humans estimated the transition probabilities between two visual or auditory stimuli in a changing environment, and reported their mean estimate and their confidence in this report. To formalize the link between both kinds of estimate and assess their accuracy in comparison to a normative reference, we derive the optimal inference strategy for our task. Our results indicate that subjects accurately track the likelihood that their inferences are correct. Learning and estimating confidence in what has been learned appear to be two intimately related abilities, suggesting that they arise from a single inference process. We show that human performance matches several properties of the optimal probabilistic inference. In particular, subjective confidence is impacted by environmental uncertainty, both at the first level (uncertainty in stimulus occurrence given the inferred stochastic characteristics) and at the second level (uncertainty due to unexpected changes in these stochastic characteristics). Confidence also increases appropriately with the number of observations within stable periods. Our results support the idea that humans possess a quantitative sense of confidence in their inferences about abstract non-sensory parameters of the environment. This ability cannot be reduced to simple heuristics, it seems instead a core property of the learning process.

## Author Summary

Learning is often accompanied by a “feeling of knowing”, a growing sense of confidence in having acquired the relevant information. Here, we formalize this introspective ability, and we evaluate its accuracy and its flexibility in the face of environmental changes that impose a revision of one’s mental model. We evaluate the hypothesis that the brain acts as a statistician that accurately tracks not only the most likely state of the environment, but also the uncertainty associated with its own inferences. We show that subjective confidence ratings varied across successive observations in tight parallel with a mathematical model of an ideal observer performing the optimal inference. Our results suggest that, during learning, the brain constantly keeps track of its own uncertainty, and that subjective confidence may derive from the learning process itself. Our results therefore suggest that subjective confidence, although currently under-explored, could provide key data to better understand learning.

## Introduction

Many animals, human adults and even human babies possess remarkable skills to cope with the pervasive uncertainty in their environment [1,2]. Learning processes are attuned to uncertainty. They enable one to capture the stochastic characteristics of the environment, as when one learns how often a probabilistic cue leads to a reward [3]. The environmental uncertainty actually occurs at several nested levels, as the stochastic characteristics themselves may also vary suddenly and without warning. The human learning is sophisticated enough to quickly adapt to such higher-order changes: the probabilities and characteristics that subjects learn are adequately fitted by statistical models [4–7]. However, in such tasks and environments flooded with uncertainty, subjects not only estimate the characteristics of the outside world, they also evaluate the degree of certainty that their estimates are accurate. This more subjective aspect of learning, the “feeling-of-knowing”, has received little attention so far. Here, we attempt to provide a formal account of this feeling and its origin.

The feeling-of-knowing, or the sense of confidence, has been primarily demonstrated in memorization tasks [8] and in perceptual decision-making tasks in humans, monkeys and rodents [9–11]. By contrast, evidence from probabilistic learning tasks is currently limited. Many learning models actually simply do not consider feeling-of-knowing as a component of the learning process. Most share a common logic, according to which each parameter of the environment is represented at any given moment by a single numerical estimate and is continuously updated based on new observations. Rescorla and Wagner suggested a simple update rule: the point estimate should be shifted in proportion of the prediction error, i.e. the extent to which the estimate deviates from the new observation [12]. Such models therefore only provide point estimates, and they are devoid of any sense of uncertainty. It has been recognized more recently that the learning rate could actually be modulated as a function of an internal estimate of the environmental uncertainty, e.g. volatility [4,6] and that learning could even be fully reset when an environmental change is detected [7]. However, the normative Bayesian approach of learning suggests that there is a principled distinction between this environmental uncertainty and the uncertainty in the internal knowledge of what has been learned [13]. We term this second kind of uncertainty, the 'inferential uncertainty'. Despite evidence that the inferential uncertainty could affect learning in humans [5], how humans perceive this uncertainty remains largely unexplored. Here, we suggest that the feeling-of-knowing, or subjective confidence, corresponds formally to the inferential uncertainty and that it derives from the inference that underpins the learning process itself.

Indeed, the fact that humans have distinct degrees in their feeling-of-knowing suggests that they do not keep track of point estimates of environmental parameters, but instead of a set of estimates, each with its own degree of plausibility. Supporting this idea, some models assume that the brain infers full probability distributions [14]. The hypothesis was initially introduced for sensory representations, but it may be extended to higher-level tasks [15–17], possibly including the learning of any numerical parameter. Following this hypothesis, learning in an uncertain world would be underpinned by a probabilistic inference that provides, not a single parameter value, but a distribution of possible values—and therefore affords an estimation of “feeling-of-knowing” based on the concentration of this inferred distribution onto a single value [18,19].

**Fig 1:**
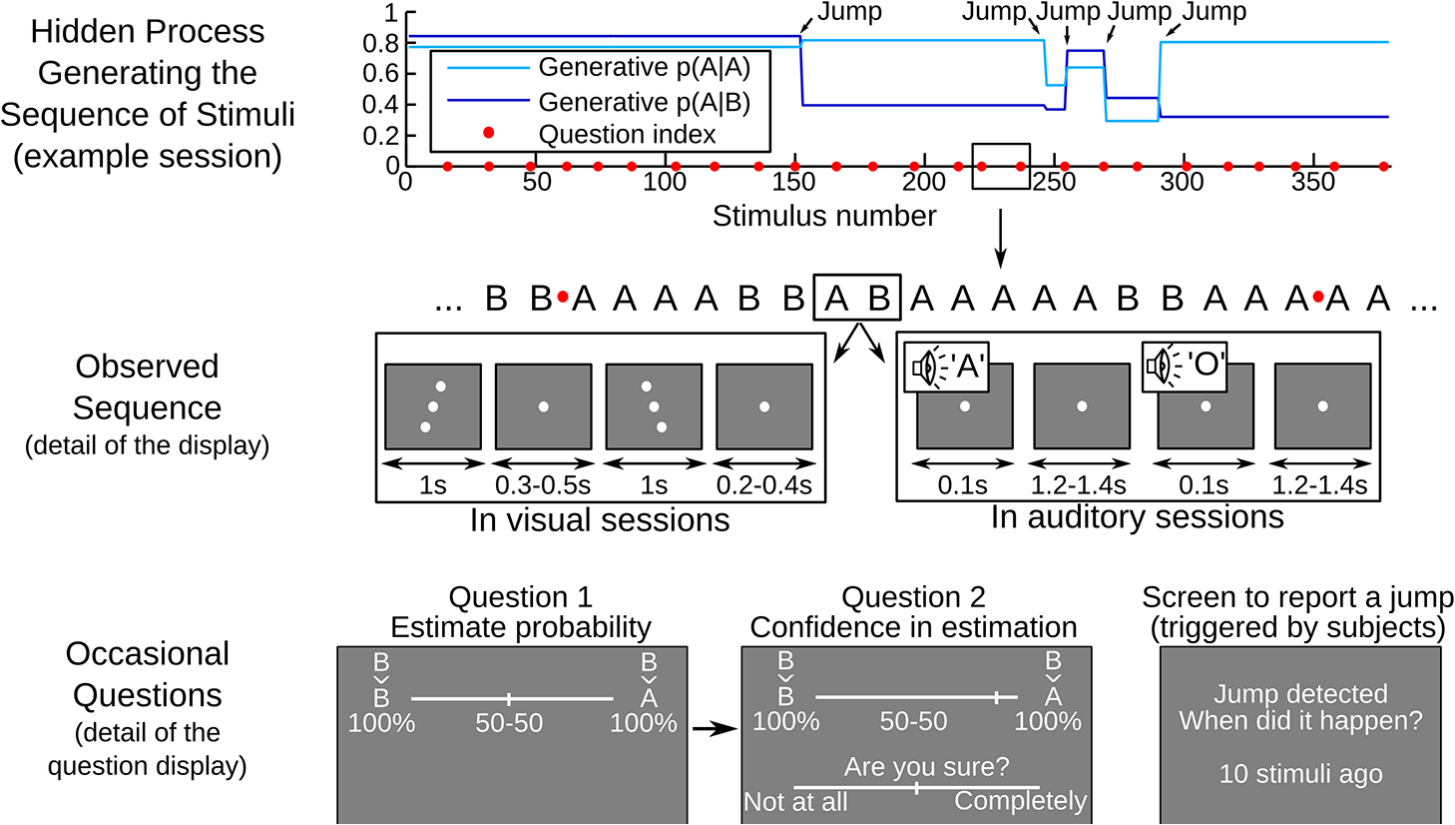
Behavioral task for the joint assessment of probability estimates and confidence. Subjects were presented with series of auditory or visual stimuli (denoted A and B) and were occasionally interrupted by questions asking for their estimate of transition probability (e.g. B→A) and their confidence in this judgment. The top graph illustrates the characteristics of the hidden process generating the sequence of stimuli in an example session: transition probabilities changed 5 times at random 'Jump' points, delimiting 6 chunks of variable length. The middle section shows a portion of the generated sequence. The actual stimuli are illustrated by gray screen-shots: in different sessions, stimuli were either visual (a line of dots tilted clockwise or anti-clockwise) or auditory (vowels 'A' or 'O' played through a loudspeaker). The sequence of stimuli was interrupted every 15 ± 3 stimuli (see red dots). At this moment, the previous stimulus (here B) was displayed and subjects indicated with a slider their estimate of the probability for the next stimulus to be A or B. In the actual display, A and B were replaced by the corresponding visual symbols or vowels. Once subjects had validated their probability estimate, they were asked to rate with a slider how confident they were in their probability estimate. Subjects also had to report on-line when they detected jumps: they could stop the sequence at any time by pressing a key to indicate how long ago the jump had supposedly occurred (see the bottom right-hand screen shot). After such reports, the stimulus sequence was resumed without feedback.

**Fig 2:**
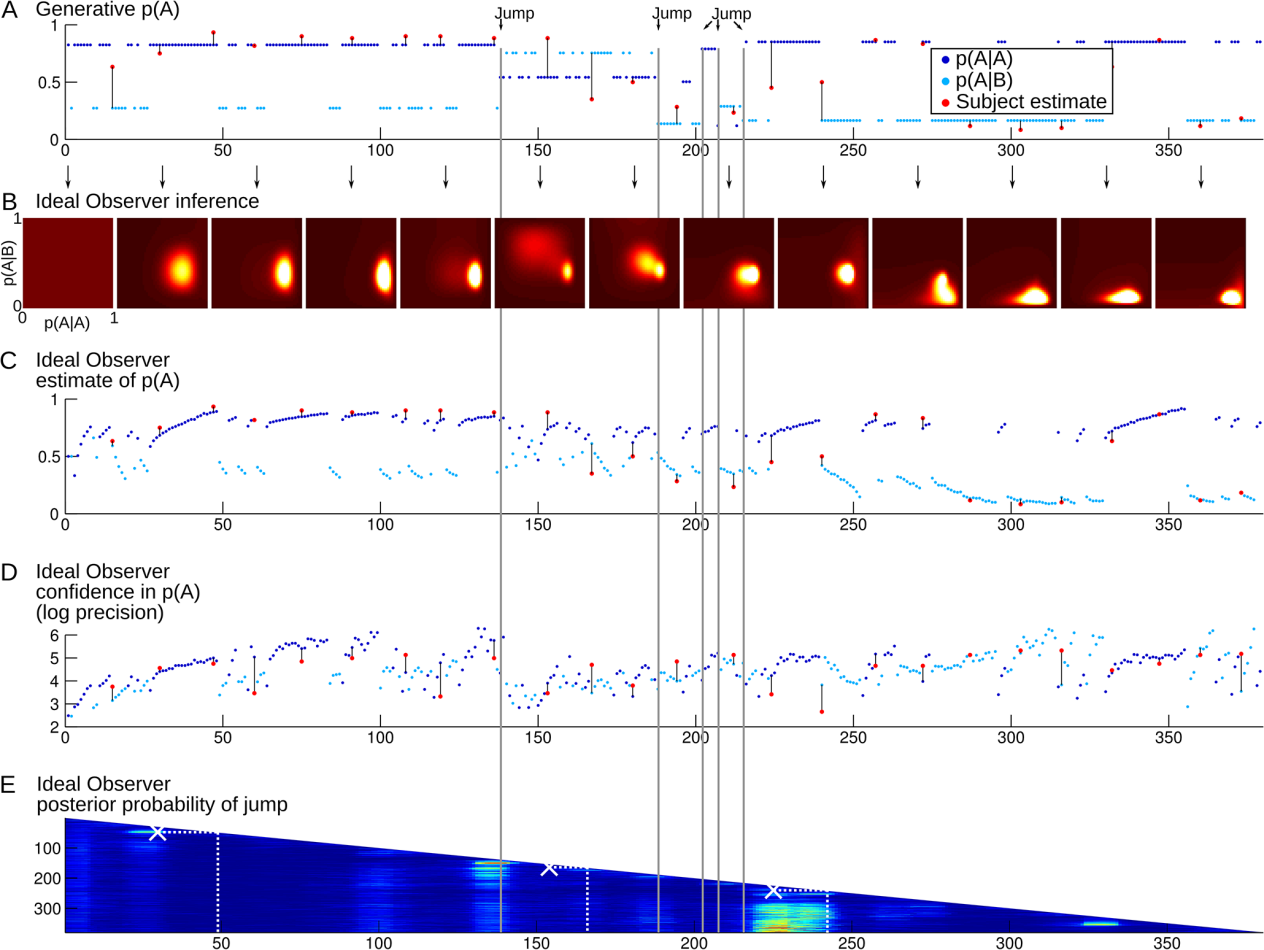
Example of the time course of a full session. *(A)* Each dot represents a stimulus in the sequence (dark blue = A, light blue = B). The position on the y-axis shows the true generative probability of having the stimulus A at a given trial, which is conditional on the preceding stimulus. The 5 changes in transition probabilities are highlighted in gray. The red dots show the subject's probability estimates that the next stimulus is A (the answer to Question 1 in Fig 1). The black lines facilitate visual comparison between the subject's estimates and the corresponding generative values. *(B)* Temporal evolution of the distribution of transition probabilities estimated by the Ideal Observer. The distribution is updated at each observation, but it is plotted only every 30 stimuli for illustration purposes. The distribution is two-dimensional: p(A|A) and p(A|B) can be read as marginal distributions along the vertical and horizontal axes. Point estimates and the related confidence levels can be read respectively as the mean and negative log variance. *(C)* Temporal evolution of Ideal Observer point estimates of the transition probabilities. The transition probabilities estimated by the Ideal Observer sometimes differ substantially from the generative ones, and better account for the subject's estimates, e.g. around stimulus 240. *(D)* Temporal evolution of the Ideal Observer confidence in the estimated transition probabilities. Confidence levels from the Ideal Observer and the subject cannot be compared directly: subjective reports were made on a qualitative bounded scale (Question 2 in Fig 1) whereas the Ideal Observer confidence in principle is not bounded. For illustration purpose, subjective confidence levels (red dots) were overlaid after adjusting their mean and variance to match those of the Ideal Observer. Several features are noteworthy: drops of confidence levels after suspicion of jumps (e.g. around stimulus 50) and a general trend for confidence to increase with the number of observations within a chunk (e.g. from stimulus 1 to 50). *(E)* Evolution of the posterior probability that a jump occurred around (±5) each stimulus of the observed sequence, as estimated by the Ideal Observer. Hotter colors denote higher probabilities. This estimation is revised after each new observation. The successive estimations result in the succession of longer and longer rows as more and more stimuli are observed in the sequence. Jumps reported by the subject are overlaid as white crosses. For instance, at stimulus 50, the subject pressed the detection key to report a jump located at stimulus 30. This detection was actually a false alarm with respect to the generative jumps, but the Ideal Observer also estimated that a jump was likely at this moment.

To test this idea, we examined whether humans can provide not only accurate estimates of environmental probabilities, but also accurate confidence ratings in those estimates. Such a finding would imply that the brain not only computes a point estimate, but also, at a minimum, the uncertainty in inferring its value, and perhaps even its full distribution. We designed a challenging probabilistic learning task with two nested levels of environmental uncertainty. Fig 1 shows how we generated the random sequences of visual or auditory stimuli and Fig 2A shows an example session. First, at any given moment, the sequence depends on two parameters: P(A|B) and P(B|A), i.e. the transition probabilities between stimuli A and B. Second, these transition probabilities themselves remain stable only for a limited time, then change abruptly to a new random value, thus delineating ‘chunks’ in the sequence separated by ‘jumps’. These jumps were aimed at inducing fluctuations in the inferential uncertainty over time. Subjects were asked to detect the jumps and, occasionally, to report their estimate of the transition probability to the next stimulus and their confidence in this estimate.

Subjective estimates of transition probabilities can be compared to the true generative probabilities. However, this comparison is not completely fair because the generative parameters are not available directly to the subject, but can only be inferred from the specific stimuli received. Furthermore, confidence is simply not a characteristic of the generative process, but solely of the inference process. This highlights the need to derive both the estimates of transition probabilities and confidence levels in a principled manner from the inference itself. We therefore compare subjects' answers with the inference generated by an Ideal Observer endowed with the mathematically optimal inference process. This normative solution formalizes the link between the inference on the one hand, and the probability estimates and confidence levels on the other. Indeed, the optimal inference returns a distribution of likelihood over the transition probabilities, given the specific stimuli received. Both a point estimate and a confidence level in this estimate can be derived from this distribution. The distribution can be averaged to obtain a single best estimate of the transition probability. Confidence should reflect how precise this estimate is: whether the distribution is spread (low confidence) or concentrated (high confidence) around this estimate. We thus formalized confidence as the precision of the distribution (its inverse variance), as previously suggested [19].

The Ideal Observer being normative, it provides a reference to assess the accuracy of the single point estimates and the fluctuations in confidence levels reported by subjects. In addition, since the Ideal Observer formalizes how single point estimates and confidence levels should derive from the inference process, it affords a series of predictions serving as tests of whether the reported estimates and confidence levels indeed derive from a common inference. And last, if confidence levels derive from an accurate inference, then they should reveal several specific properties of this efficient inference system.

## Results

### Subjects accurately detect changes in the generative process

We first asked whether subjects could detect when the characteristics of the sequence changed suddenly. We assessed the accuracy of their detection in comparison to the actual position of jumps with a Receiver Operative Characteristic analysis. Subjects reported more jumps when transition probabilities were indeed changing (hit) than when they were stable (false alarm): the difference of hit minus false alarm rates was 0.23 (standard error = ± 0.03; t-test against 0: p<10^–5^). To show that this difference is positive not because of chance, but instead because the detection is based on the actual evidence provided by the observed sequence, we used a more conservative test. Comparison with surrogate data indicates that the observed difference between the hit and false alarm rates is significantly higher than expected from a random detection process (p<0.01, see Methods).

**Fig 3:**
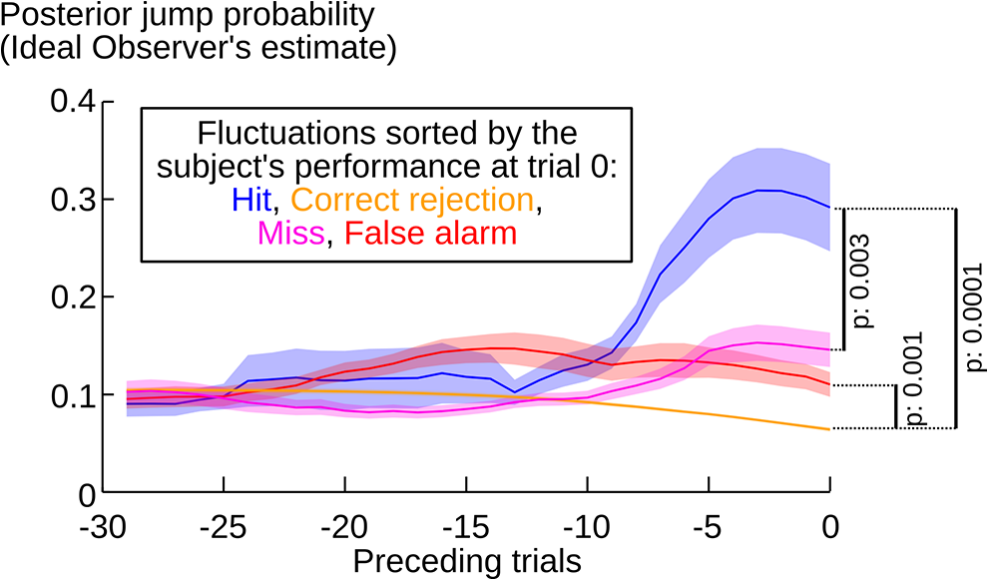
Accuracy of the subjects' jump detections. Subjects’ jump detections followed the fluctuations in jump likelihood provided by the sequence of stimuli. Each trial was sorted into four categories (hit, miss, correct rejection and false alarm), based on the comparison between the subjective jump detection and the actual position of jumps in the sequence. This sorting was used as a reference (trial 0) to examine the fluctuations in the posterior jump probability estimated by the Ideal Observer over the preceding trials. P-values correspond to two-tailed paired t-tests at trial 0. Solid lines and error shadings correspond to mean ± sem over subjects.

Although the detection of jumps by subjects is better than chance, it is not perfect: some jumps were missed, and some others falsely reported. However, some of these errors precisely further demonstrate that subjects based their detection on the actual level of evidence received. Indeed, in principle, not all jumps can be detected equally easily: for instance, when changes in transition probabilities are small and frequent, the sequence may not provide enough evidence for the presence of each jump. The Ideal Observer provides a principled way of quantifying the likelihood of a jump at each position in the observed sequence. We tested whether subjects are sensitive to such fluctuations in evidence by analyzing their errors (misses and false alarms) from the Ideal Observer perspective. A significant difference in jump likelihood at the time of the subjects' Hits vs. Misses (p = 0.003) indicates that subjects were more likely to miss a jump when the apparent jump likelihood was misleadingly low. Similarly, a difference between False Alarm vs. Correct Rejection (p = 0.001) reveals that the subjects' false alarms were more likely to occur when jump likelihood was high (see Fig 3).

Altogether, these results indicate that subjects partially managed to track the jumps in the objective generative process, and their responses give evidence of an efficient statistical use of the available information.

### The estimates of transition probabilities are accurate despite the instability of the generative process

**Fig 4:**
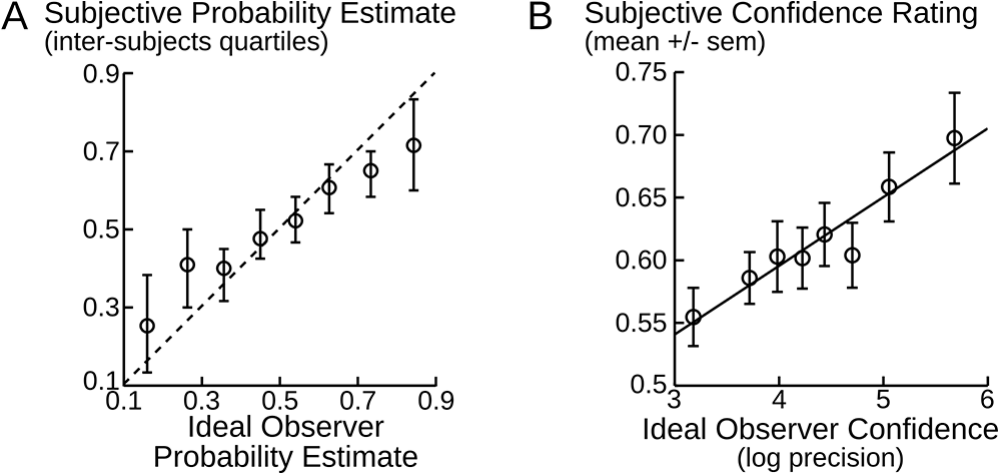
Accuracy of probability estimates and confidence. *(A)* Estimated probability that the next stimulus is A plotted against the Ideal Observer estimate. These probability estimates correspond to the transition probabilities p(A|A) or p(A|B), depending on whether the previous stimulus was A or B; both are pooled together. The dotted line corresponds to the identity. Error-bars and dots are the 75%, 50% and 25% percentiles across subjects. *(B)* Subjective confidence plotted against the Ideal Observer confidence. The steps of the subjective confidence scale were coded such that 0 corresponds to 'Not at all sure' and 1 to 'completely sure'. The Ideal Observer confidence is summarized as the log precision,-log(σ^2^), with σ² the variance of the estimated transition probability distribution. The fitted line is the average of the linear fits performed at the subject level. In A & B, equally-filled data bins were formed along the horizontal axis because the sequence of stimuli (and hence, estimates that can be inferred) differed across participants. Bins are used only for visualization and not for data analysis.

We next examined whether subjects could estimate the characteristics of the sequence despite their unpredictable changes in time. The sequence was paused every 12 to 18 stimuli and participants were asked to report the probability that the next stimulus would be A or B. Subjects’ responses were correlated across trials with the true generative probabilities (t_17_ = 8.8, p<10^–7^), indicating that subjects' probability estimates, although imperfect, consistently followed the generative probabilities. The deviations could reflect that the transition probabilities are inferred from the specific and limited amount of stimuli received. We therefore compared the subjects’ estimates of transition probabilities with the optimal values that could be inferred from the data, i.e. the parameter estimates inferred by the Ideal Observer. The subjects' responses were tightly correlated with the optimally inferred probabilities (t_17_ = 8.5, p<10^–6^, see Fig 4A). When both predictors were included in a multiple linear regression, significantly higher regression weights were found for the optimal estimates than for the generative values (paired difference of weights: t_17_ = 4.6, p<10^–3^).

Given that the Ideal Observer and the subjects are both asked to estimate a probability, we can not only test whether their estimates are correlated, but also whether they are identical. Fig 4A reveals a remarkable match, although somehow imperfect: the observed slope is actually slightly below the identity. This deviation could reflect the distortion of subjective probabilities classically reported [20]. However, this pattern could also reflect differences in accuracy across trials and subjects. Indeed, the average of ideal estimates should be perfectly aligned on the diagonal, but the average of random estimates would form a flat line at 0.5; therefore a mixture of both should result in an intermediate slope. Supporting this view, the inspection of individual data revealed that the regression slopes were significantly larger than 0 in most subjects (p>0.009 for 16 out of 18 subjects), but they were significantly equal to 1 in only 3 subjects (in these subjects, Bayes factor > 9, see [21] for the computation of this 'Bayesian t-test').

Together, these results show that subjects were able to infer the transition probabilities generating the observed sequence of stimuli despite their sudden changes in time. Not surprisingly, subjects were outperformed by the Ideal Observer endowed with the best inference scheme. However, the comparison to the optimum reveals a remarkable accuracy of the subjects' estimates.

### Confidence judgments fluctuate accurately

Subjects were also asked to rate their confidence in their probability estimates. They provided confidence ratings on a bounded qualitative continuum (see Fig 1). The absolute position of a given 'feeling-of-knowing' on this continuum is a matter of subjective representation, not a property of the inference process. However, if the confidence judgment reflects the certainty of the inferred probability estimate, then distinct confidence ratings should correspond systematically to distinct levels of evidence. Therefore, we assessed the accuracy of the fluctuations in confidence judgment with a regression against a principled measure of the level of evidence. Again, we used the Ideal Observer to this end. Intuitively, confidence should be high if and only if the estimated distribution of transition probability is concentrated on the reported value. This corresponds formally to the notion of precision, the inverse variance of the estimated distribution. Thus, we defined the Ideal Observer confidence as the negative log of the variance of the distribution. We used the log scale because it is the natural space for variance [22]. Note that the log variance and log standard deviation are strictly proportional, therefore the choice of one or the other provides the exact same significance levels in the regression analyses. We found a strong positive correlation between this principled measure of confidence and the subjective confidence (t_17_ = 3.94, p = 0.001; see Fig 4B).

In addition, given that the experiment presented visual and auditory stimuli in separate blocks, we checked the robustness of the previous results by testing each modality separately. The regression of subjective estimates against the Ideal Observer was significant within each modality (for probability estimates: both p<10^–5^; for confidence: both p<0.004). Interestingly, these regression weights were positively correlated between modalities (for probability estimates, Pearson ρ_16_: 0.55, p = 0.017, for confidence ρ_16_: 0.59, p = 0.010), supporting the idea that inferential capabilities vary between observers and are not tied to one modality but instead characterize a supramodal level of processing.

Another way to evaluate the accuracy of confidence judgments is to ask whether they predict performance. Computing confidence can be useful when it serves as a proxy for the accuracy of performance—high subjective confidence should predict an objectively low rate of errors. We verified that this is true for the normative Ideal Observer: across trials, the magnitude of the error separating the Ideal Observer estimates from the true generative probabilities was negatively correlated with the Ideal Observer confidence (t_17_ = -8.67, p<10^–6^). Crucially, a similar relationship linked the subjects’ confidence with the objective error in their probability estimates (t_17_ = -2.27 p = 0.037). We used simulations to check that this link derives from the normative nature of the subjects' estimates and not from biases in their probability estimates or confidence ratings. We used three separate simulations to reassign randomly one variable (probability estimates, confidence ratings or true generative probabilities), while keeping the two others unaffected. Each simulation disrupts specific links between the generative probabilities and the subjective estimates to capture potential response biases. The simulations showed that the negative relationship observed between confidence and objective error is unlikely to emerge by chance on the sole basis of response biases (all p<0.019).

Altogether, the findings indicate that confidence estimation is accurate: it relates linearly to the principled inference made by the Ideal Observer, and it is also correlated with objective performance.

### Links between probability estimates and confidence ratings suggesting a common inference process

Our hypothesis is that estimates of transition probabilities and confidence ratings jointly derive from a single inference process. In other words, there is a common substrate for both estimates. An alternative hypothesis would be that confidence ratings are derived from the estimates of transition probabilities. Our hypothesis leads to several testable predictions that also rule out the alternative.

**Fig 5:**
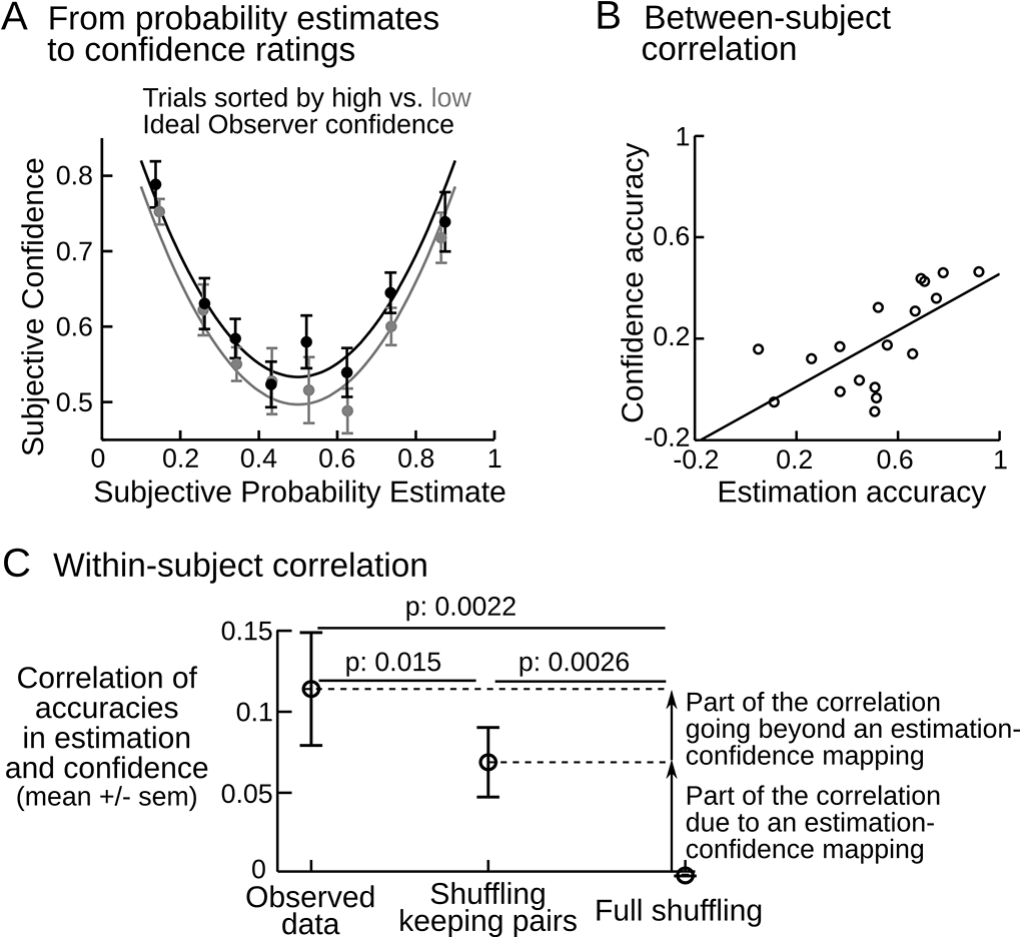
Evidence that probability estimates and confidence derive from a single process. *(A)* Subjective confidence is higher for extreme estimates of transition probabilities. The fitted lines correspond to the average of the quadratic fits performed at the subject level: confidence ~ constant + (probability estimate-0.5)^2^. Trials were sorted by subjective probability estimates and, within each bin, into high and low Ideal Observer confidence according to a median split. Equally-filled bins were used for data visualization, not for data analysis. *(B)* The accuracies of probability estimates and confidence ratings are correlated across subjects. The accuracy of probability estimates was computed *per subject* as the correlation (across trials) of the subject's and the Ideal Observer's estimates. The same logic was used for confidence. One dot corresponds to one subject. *(C)* The link between probability estimates and confidence ratings goes beyond any mapping. Within each subject, we computed the correlation across trials between accuracies in probability estimates and confidence ratings. The accuracy of probability estimates was computed *at the trial level* as the distance between the subject's and the Ideal Observer's estimates. The same logic was used for confidence. The observed results are contrasted to two ways of shuffling the data (p-values are from one-tailed t-test, see Methods).

We predict that probability estimates and confidence ratings should be partly related: when information is scarce, the optimal default estimate for transition probability is around 0.5 and confidence is low. Extreme estimates (toward 0 or 1) are achieved only when there is substantial evidence and hence when confidence is high. Confidence should thus increase when the probability estimates depart from 0.5: this is a fundamental and inescapable property of probabilistic reasoning. The Ideal Observer estimates robustly showed this U-shape pattern (quadratic weight: t_17_ = 16.1, p<10^–11^), and so did our subjects (t_17_ = 9.77, p<10^–7^). This effect was actually significant within every subject (all p<0.0025). However, we also predict that this U-shape relationship should be only partial, since in principle, one may be more or less confident in any probability estimate, depending on the number of observations that support it. To illustrate this property, we binned the participants' confidence ratings by their subjective probability estimates, and within each bin, we then sorted trials by high and low Ideal Observer confidence with a median split (Fig 5A). Subjective confidence reflected the Ideal Observer confidence on top of the general U-shape pattern. To quantify this additional effect, we performed a multiple regression of subjective confidence, without binning, including as predictors both the subject's U-shape transformed probability estimates and the optimal confidence. The data revealed that the Ideal Observer confidence indeed captures aspects of subjective confidence (t_17_ = 3.12, p = 0.006) that are not accounted for solely by a quadratic effect of probability estimates.

In our experiment, subjects reported their probability and confidence estimates sequentially. We therefore ran a control experiment to check that the nested relationship between probability estimates and confidence ratings (as shown in Fig 5A) is a general property of human reasoning which cannot be attributed to sequential reporting. New subjects performed a variant of the task in which the first question about the probability estimate was omitted (see Methods). Subjective confidence rating still followed the Ideal Observer confidence (t_20_ = 7.00, p<10^–5^). As expected from a probabilistic inference, subjective confidence also showed a quadratic effect of the optimally inferred probability (t_20_ = 6.97, p<10^–5^). In addition, subjective confidence still co-varied with the Ideal Observer confidence on top of the quadratic effect of the optimal probability (multiple regression: t_20_ = 3.01, p = 0.007, t_20_ = 5.13, p<10^–5^ respectively).

Our main experiment enables to further test the predictions of our hypothesis concerning the common origin of probability and confidence judgments. If probability estimates and confidence ratings both derive from the same inference, then we also expect that subjects who perform the inference accurately should perform accurately in *both* estimating probabilities and rating confidence. We defined how accurate subjects were in estimating probabilities and rating confidence with respect to the Ideal Observer. In both cases, accuracy was summarized as the correlation coefficient between the subjects’ response and the optimal response. We found a positive correlation across subjects between the accuracies of probability estimates and confidence ratings (Pearson ρ_16_ = 0.67, p = 0.002, Fig 5B). We also tested whether this correlation was significant *within* subjects. On each trial, we computed the accuracy of probability estimates (or confidence ratings) as the distance between the Ideal Observer and the subject's responses (see Methods). Again, the two accuracies were significantly correlated across trials (t_17_ = 3.27, p = 0.005). Note that these correlations are also consistent with the alternative hypothesis that confidence ratings are derived from probability estimates. However, the within-subject data disprove this alternative. Indeed, we controlled that the within-subject correlation we found is not confounded by an effect of an estimation-to-confidence mapping (be it quadratic or not) by comparison with two shuffled data sets (see Methods and Fig 5C).

Altogether, these results show that probability estimates and confidence ratings are likely to derive from a common inference. In particular, the accurate confidence ratings reflect additional features of the inference that are not reflected in the probability estimates.

### Fluctuations in confidence levels reflect the accuracy of the inference on a trial-by-trial basis

We now examine whether the data provide cues as to how confidence is computed. The inference should use the incoming data to constantly update an internal model of the hidden process that could have generated the observed sequence of stimuli. There are normative principles ruling this update process. Therefore, any efficient algorithm should have specific characteristics. We show that confidence ratings reveal three properties expected from an efficient information processing system.

**Fig 6:**
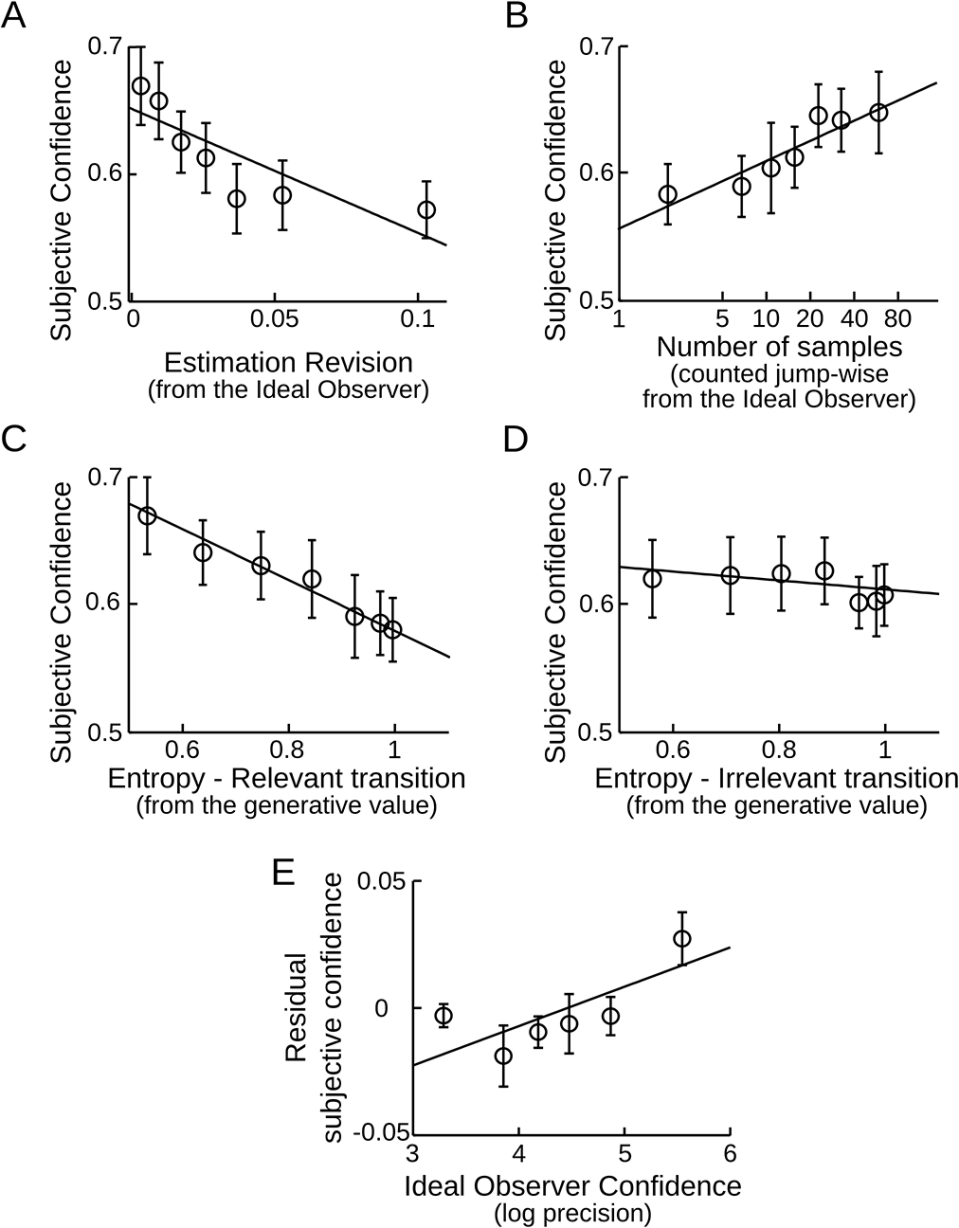
Subjective confidence is updated appropriately on a trial-by-trial basis. *(A)* Confidence varies inversely with model revision. The revision of probability estimates corresponds to the shift (absolute difference) in transition probabilities estimated by the Ideal Observer, between two consecutive observations of this transition. *(B)* Confidence increases when there is more information. Mathematically, confidence should increase linearly with the log-number of samples within stable periods; thus a log-scale is used to plot subjective confidence. The sample count was reset each time the Ideal Observer detects a new jump. *(C*, *D)* Confidence is reduced when transitions between stimuli are less predictable. The entropy reflects how unpredictable the next stimulus is based on the generative transition probability: p(A|A) or p(A|B). If the stimulus preceding the question is A, the relevant transition entropy is determined by p(A|A). By contrast p(A|B) is irrelevant. *(E)* Evidence that subjective confidence estimation goes beyond all of the above factors taken together. A multiple regression including the factors in panels A to D was used to compute the residual subjective confidence, which was then correlated with the Ideal Observer confidence. In all plots error-bars give the inter-subject mean ± s.e.m; the fitted line is the average of the linear fits performed at the subject level. Bins are used only for visualization and not for data analysis.

First, whenever the probability estimates change a lot, indicating a severe revision of the internal model (for instance, after a jump), then confidence should be low; conversely, when estimates are stable, confidence in the seemingly 'good' value should be high. Questions being asked only occasionally to the subjects, the subjective model revision cannot be estimated from their reports. Instead, we estimated the degree of model revision from the Ideal Observer. This ensures in addition that subjective confidence is regressed against a normative estimate in every subject. We observed the predicted negative correlation between subjective confidence and the amount of revision in the probability estimates relative to the previous observation (t_17_ = 3.67, p = 0.002; Fig 6A), indicating that subjective confidence tracks the revision of an internal model.

Second, the number of data samples accumulated since the last detected jump should affect the level of confidence: more samples should lead to more precise estimations. We counted the cumulative number of samples between the optimally detected jumps. As predicted, we observed a positive correlation between subjective confidence and the number of samples since the last jump (t_17_ = 3.51, p = 0.003; Fig 6B), indicating that subjective confidence increases with the accumulation of evidence. Again, using the Ideal Observer to estimate the number of samples in the current chunk provides a normative comparison across subjects. Instead, using the subjects' jump detection entangles several factors, e.g. whether subjects are accurate and conservative in reporting jumps. The same analysis based on the subjects' jump detection however also revealed a positive correlation (t_17_ = 2.26, p = 0.037).

Third, confidence should be lower when the estimation of the model is made more difficult by decreasing the predictability of the sequence. Formally, the unpredictability of a sequence is characterized within a chunk by the entropy of the generative transition probabilities: it is maximal when the transition probability is 0.5 and it decreases as the transition probability goes toward 0 or 1. Note that we quantify here the generative environmental uncertainty, not its subjective estimate (as in Fig 5A). We therefore examined if confidence was negatively correlated with this entropy. As predicted, a negative correlation was observed (t_17_ = -5.58, p<10^–4^; Fig 6C). As a control, we examined if a similar effect occurred when computing the entropy of the other, currently irrelevant transition probability (transition from the stimulus which was not presented on the previous trial). No significant effect was found (t_17_ = -0.76, p = 0.50; Fig 6D). The results therefore indicate that subjects keep a distinct record of the confidence attached to each of the two transition probabilities that they are asked to estimate.

We checked that the results presented in Fig 6 survive correction for multiple comparisons and partial correlations by including the four regressors into a multiple regression of confidence levels. The three factors of interest were still significant (amount of model revision needed: p = 0.006; number of samples received: p = 0.042; entropy of the relevant transition: p = 10^–5^, and not the irrelevant one: p = 0.3). We also confirmed that these results coincide with the normative theory by running the same analysis on the Ideal Observer confidence (effect of the 3 factors of interest: |t_17_|>8.7, p<10^–7^, no effect of the irrelevant transition entropy: t_17_ = -0.3, p>0.7). These results support the idea that confidence ratings derive from a rational process that approximates the optimal probabilistic inference.

### The accuracy of confidence resists simple heuristics and suggests instead a probabilistic inference

Our hypothesis is that confidence and probability estimates both derive from the probabilistic inference itself. An alternative is that subjective confidence is derived independently with a valid heuristic [23,24]. In the current experiment, the probability estimate for instance is a rational cue for confidence: as discussed with Fig 5A, there is a strong and principled correlation between confidence and how much the probability estimate departs from 0.5. However, we showed (in Fig 5A and 5C) that the accuracy of confidence judgment goes beyond this kind of mapping. This therefore precludes that subjective confidence derives only from a heuristic based on the probability estimate. An example of such heuristic would be to count the number of correctly predicted stimuli in the immediately preceding trials to determine a confidence level.

We then showed that confidence is also systematically impacted by the entropy of the generative transition probability, the amount of samples accumulated in the current chunk and the degree of revision of the probability estimates (Fig 6). At a minimum, these results imply that confidence arises from a sophisticated heuristic that combines the above factors. However, we can prove here that human confidence ratings are more accurate than such a heuristic would predict: even after regressing out the effect of the above factors, the residual subjective confidence still co-varied significantly with the Ideal Observer confidence (t_17_ = 2.89, p = 0.01, Fig 6E).

What additional features of the inference process could explain this finding? In deriving the “number of samples” heuristic, we assumed that subjects discretize the incoming sequence into discrete chunks separated by jumps, and that this process allows them to track how much evidence they received since the last jump. This heuristic is suboptimal, however: the optimal inference avoids any discrete decision, but computes with the full probability distribution that a jump occurred at any moment, and uses it to weight recent evidence. To evaluate whether human subjects integrate jump likelihood into their confidence estimates, we computed, on each trial, the current uncertainty on the location of the last jump. We quantified it as the variance of the current chunk length estimated by the Ideal Observer, normalized by its mean value (over similar positions in the sequence across sessions and subjects) so that values higher than 1 indicated that it was less clear than average when the last jump occurred. Subjective confidence correlated negatively with this uncertainty on jump location (t_17_ = -3.12, p = 0.006), exactly as expected from a normative viewpoint (same analysis with Ideal Observer instead of subjective confidence: t_17_ = -5.71, p<10^–4^). It therefore seems that subjects are able to factor an estimate of jump probability in their confidence judgments. Altogether, these results suggest that the inference underpinning learning in this task is a probabilistic computation.

## Discussion

We present an in-depth analysis of how humans acquire explicit knowledge and meta-knowledge of transition probabilities in an unstable environment. Our results demonstrate that subjects use the available stochastic evidence to learn about the incoming sequence: their estimates of two transition probabilities P(A|B) and P(B|A) accurately track the true generative values. Most importantly, by asking subjects to systematically rate their confidence in those estimates, we show that humans can accurately evaluate the uncertainties associated with each piece of information that they acquire. This sense of confidence, which affords a quantitative and explicit report, is available in a modality independent manner for both visual and auditory sequences, and it closely tracks the fluctuations in uncertainty that characterize an accurate probabilistic inference process.

### Distinct forms of uncertainty

Several classifications of uncertainties have been proposed [25]. Our distinction between environmental and inferential uncertainties is close to Kahneman and Tsersky’s [23] classical division of external uncertainty (stochastic nature of the environment) versus internal uncertainty (state of knowledge). A similar distinction is also made in recent computational works, e.g. in [13], the environmental uncertainty would correspond to the 'risk' and 'unexpected uncertainty', the inferential uncertainty to 'estimation uncertainty'; in [5] a similar distinction is made. Internal uncertainty is sometimes called ambiguity, in particular in economics, when it characterizes the absence of knowledge [25,26]. Our terminology (environmental vs. inferential uncertainties), stresses that these two kinds of uncertainties differ in their epistemic nature. By operationalizing this distinction, our study revealed how they are only partially related. We built upon previous paradigms that manipulated environmental uncertainty [4,7] in order to induce frequent variations in inferential uncertainty. We showed how a first-order environmental uncertainty (probabilistic transitions between stimuli) increases the inferential uncertainty, and how a second-order environmental uncertainty (unexpected changes in these transition probabilities) produces additional fluctuations in inferential uncertainty over time. The fact that environmental and inferential uncertainties are only partly related is particularly salient in our task when a transition probability is 0.5. Such probability produces the least predictable outcomes (high environment uncertainty) and a precise estimation of this probability needs more samples than any other probabilities (hence, a high inferential uncertainty). However, with a large number of observations, one can get quite confident that the outcomes are indeed completely unpredictable. All these effects were observed in a normative Ideal Observer model, and subjects' confidence faithfully tracked ideal-observed confidence. Thus, human adults possess sophisticated mechanisms for tracking their inferential uncertainty.

Juslin & Olson [27] made a different distinction, separating Brunswikian uncertainty, independent from us and in that sense 'external', and Thurstonian uncertainty, due to the imprecision of our information-processing systems. While Thurstonian uncertainty may have contributed to the small deviations that we observed between subjective confidence and the optimal observer, we stress here that learners are uncertain, not only because they are faulty, but primarily because inference from stochastic inputs is by essence uncertain. The Ideal Observer quantifies this irreducible level of inferential uncertainty that any learner must face in our task. It is an open question whether and how humans may combine this core inferential uncertainty with the additional uncertainty arising from their cognitive limitations.

### New perspectives on confidence

Broadly defined, confidence indexes a degree of belief in a particular prediction, estimation or inference [19,23,25]. What confidence is about may thus vary drastically, from mere detection (feeling of visibility, e.g. [28]), to accuracy in perceptual tasks [9,10,29], in memory retrieval [8], or in response to general-knowledge questions [30,31]. Mathematical concepts clarify how the present work differs from these previous studies. In most studies, confidence can be formalized as the likelihood of some binary variable e.g. the posterior probability that a response is correct/incorrect, a stimulus is seen/unseen, etc. [9]. By contrast, here we investigated confidence in a continuous numerical quantity (the inferred transition probability), so that a principled and natural formalization for the strength of evidence is, as suggested previously [19], the precision of this variable (its inverse variance). This computational distinction, in comparison with most previous studies, entails a noticeable difference in practice. In typical binary decision tasks, the accuracy of subjective confidence is estimated by comparison with the actual performance of the subject. This estimation may be more or less susceptible to biases [32]. In our task, confidence is defined as the precision of the variable inferred, and is therefore amenable to a principled quantification with the Ideal Observer. Therefore here, the accuracy of subjective confidence can be estimated by comparison with this optimal confidence. Crucially, this estimation is independent from the performance in the primary estimation task, which may even remain unknown to the experimenter.

One could disagree with our particular formalization of confidence, and suggest alternative mathematical quantities such as the inverse variance (not its log, as we did), or the posterior probability of the mean or of the maximum of the inferred distribution, or the entropy of this posterior distribution. All these metrics roughly quantify the same notion: they are highly correlated with the one we used, and running the analyses with these other metrics led to similar (although less significant) results. The tight correlation between the ideal-observer precision and human subjective confidence therefore strongly suggests that humans possess a remarkable capacity to extract and use probabilistic information.

We assessed the accuracy of the subjective precision estimates based on their relative variations between trials. The metacognition literature however makes a classical distinction between whether the accuracy of confidence is only relative or also absolute [31]. Absolute confidence levels, and thus the identity between the subjective and the optimal levels, cannot be investigated in our design: indeed, mapping confidence onto a qualitative scale is subjective, not principled. Subjects may produce absolute confidence measures for binary variables, e.g. they may estimate the fraction of correct or seen trials, but asking them a numeric estimate of subjective precision seemed too difficult, which is why we resorted to a qualitative confidence scale. This aspect of our study leaves open the question of whether there is an internal scale for precision that could be sufficiently calibrated to be transferred between tasks [33] or even individuals [34], as previously shown for binary judgments.

Our estimation of the accuracy of subjective confidence relies on a comparison with an Ideal Observer. However, the literature on the perception of probabilities have evidenced frequent deviations from optimality, e.g. the over and under estimation of small and large probabilities [35,36], and a bias toward the detection of alternation vs. repetition [37,38]. Whether adjusting the Ideal Observer to these biases could provide a tighter fit to subjective data is an open issue and a matter for further research. Different options are available to include these biases in the ideal observer model. One possibility is that only the report of the probability is distorted. In that case, the inference, and hence the confidence levels, would remain unaffected. By contrast, the bias could affect a particular component of the inference itself. Potential targets for such distortions include (1) the likelihood of the current observation given some inferred probability estimate, which serves to update the posterior knowledge; (2) the posterior estimate itself, which serves to evaluate the likelihood of future observations; (3) the prior about the generative probabilities, which biases the inference at the beginning of each new sequence, but also at any time a jump in probabilities is suspected. These different potential sources of bias may result in quantitative differences in confidence levels, which could help to arbitrate between these scenarios.

### Constraints on models of the learning process

Our results reveal some characteristics of the computation of confidence in humans. One possibility is that second-order estimates occur independently from the first-order estimates, by relying on indirect cues or heuristics such as reaction time in the first-order task [23,24]. However, several aspects of our results contradict this view. First, the sophisticated heuristics we tested did not fully account for confidence reports; similar results were reported in the perceptual domain [39]. Second, the accuracies of the first and the second-order estimates were tightly correlated across trials and subjects which contradicts that confidence levels occur independently.

The alternative view is that first and second-order processes are related, e.g. the second-order process relies on a readout of the same single-trial inferential data available to the first-order process [40–42]. Signal detection theory formalized this readout process in perceptual decisions, postulating that the second-order estimate corresponds to a statistical quantity (d-prime) characterizing the first-order process [32]. Our hypothesis extends this idea to the learning domain: learning could be supported by a probabilistic inference [17,43], resulting in a posterior distribution whose mean and precision would yield, respectively, the first-order and second-order estimates.

The terms first-order and second-order estimates may indeed be unfortunate, as they suggest a sequential process. It is in fact an open issue whether the primary response and the confidence in this response arise in parallel or serially, and from a single brain circuit or not [11,40]. Parallel extraction by distinct circuits could account for the fact that confidence and performance are often correlated, but still dissociable [44,45], for instance in situations of speeded judgment [29], overconfidence [46], or when the accuracy of confidence is impaired while performance is preserved.

By revealing some characteristics of the computation of confidence, our results may reveal some characteristics of the learning process itself. Indeed, if both the learned estimates and the assigned subjective confidence levels derive from the same inference, then investigating subjective confidence could provide critical insights on the learning process. It should be the case if subjective confidence levels reveal something more than what the learned estimates already reveal by themselves. We showed that it is the case: the accuracy of subjective confidence cannot be reduced to the accuracy of the learned estimates. This implies that the classic view of learning, exemplified by the Rescorla Wagner rule, according to which learning simply consists in updating parameter estimates, does not suffice—the brain also keeps track of the uncertainty associated with each value. Recent computational works have already started to revisit this classic learning model so as to incorporate notions of uncertainty [5,13]. Our results emphasize the need to investigate confidence as part of the learning algorithm. Future work should determine whether learning relies on simplified computations involving only summary statistics such as mean and variance [5], on sampling schemes [17,47], or on full computations over distributions [15].

## Methods

### Ethics statement

The study was approved by the local Ethics Committee (CPP n°08–021 Ile de France VII) and participants gave their informed written consent prior to participating.

### Task and participants

18 participants (9 females, mean age 23, sem: 0.74) were recruited by public advertisement. The task was delivered on a laptop using Matlab (Version R2013a) and PsychToolBox (Version 3.0.11). The experiment was divided into 4 blocks, each presenting a sequence of 380 stimuli (denoted A and B). On alternated blocks, A and B were either auditory or visual stimuli perceived without ambiguity, see Fig 1 for a description and the timing. A fixation dot separated the visual stimuli and remained present during the auditory blocks. The modality used in the first block was counterbalanced over subjects.

The sequence was generated randomly based on predefined transition probabilities between stimuli, e.g. an 80% chance that A is followed by A and a 30% chance that B is followed by A. These values are thus called 'generative transition probabilities'. The sequence was structured into chunks: transition probabilities were constant within chunks and changed from one chunk to the next at so-called 'jumps'. Chunk lengths were sampled from a geometric distribution, with an average chunk length of 75 stimuli. To avoid blocks without jumps, chunks longer than 300 stimuli were discarded. In each chunk, transition probabilities were sampled independently and uniformly in the 0.1–0.9 interval, with the constraint that, for at least one of the two transition probabilities, the change in odd ratio p/(1-p) relatively to the previous chunk should be at least 4. The sequence was paused occasionally (every 15 stimuli, with a jitter of ± 1, 2 or 3 stimuli) to ask subjects about their probability estimates and confidence (see Fig 1). Probing subjects more often would have provided more information on their internal estimates; however it would also have disrupted more their effort to integrate serial observations, which is critical to estimate *transition* probabilities. Asking every 15 stimuli is thus a compromise. The raw data are provided as Supporting Information (S1 Dataset, see S1 Text for a description).

20 participants (12 females, mean age 25, sem: 0.76) were recruited for the control experiment. The key difference compared to the main task was that subjects were only asked the confidence question. The other task parameters were identical, excepted a minor modification: subjects used a four-step scale instead of a continuous scale to report their confidence level. Subjects first performed one session of the main experiment which served as training. Then, they performed four sessions of the modified task.

### Instructions and training

All participants received detailed explanation about how the sequences are generated. An interactive display made intuitive the notions of transition probabilities, jumps and randomness. Transition probabilities were framed as state-dependent probabilities: e.g. if the current stimulus is A, there is an 80% chance that it is repeated and a 20% chance that it changes for B. For each state ('after A' and 'after B') these contingencies were presented as pie-charts. Random sampling from these contingencies was illustrated as a 'wheel of fortune': a ball moved around the pie chart, with decreasing speed, and the final position of the ball determined the next stimulus (A or B). Participants could repeat this process and simulate a sequence of stimuli until they felt familiar with the generative process. To introduce the concept of jump, a dedicated key press triggered a change in the pie-chart (hence, in transition probabilities).

During the task, subjects were instructed to report jumps. They could press a key at any moment to pause the sequence and access the bottom right-hand screen shown in Fig 1. By adjusting the counter displayed, they specified when the jump occurred (e.g. '13 stimuli ago'). It was made clear that 1) the estimation and confidence questions would be prompted automatically, 2) the occurrence of questions and jumps was predefined and independent so that it was unlikely that a question prompt would coincide with a jump and 3) answers in the task had no impact on the actual generative transition probabilities.

### Analysis of jump detection

We used two methods to analyze the accuracy of jump detection. The first is the classic approach of the Receiver Operating Characteristic (ROC): the reported jumps were compared to the actual, generative jumps. The second approach is a follow-up of the ROC analysis, benefiting from the Ideal Observer perspective: the binary subjective reports (there is a jump vs. there is not) were compared with the continuous, normative posterior probability of a jump.

For both approaches, we sorted the subjects' responses into hits and false alarms. Given the stochastic nature of the task, it is difficult to detect exactly when a jump occurred. Consider for instance the sequence:

A_1_ B_2_ A_3_ A_4_ A_5_ A_6_ B_7_ A_8_ A_9_ *A*_*10*_ *B*_*11*_ *B*_*12*_ *B*_*13*_ *B*_*14*_ *A*_*15*_ *B*_*16*_ *B*_*17*_ *B*_*18*_

Subscripts indicate stimulus position and the italic font indicates the second chunk. These chunks were generated from the following transition probabilities: low for AB and high for BA from stimulus 1 to 9; high for AB and low for BA from stimulus 10 to 17. The true generative jump occurred at stimulus 10, yet it seems more likely to have occurred at stimulus 11: A_9_A_10_ better fits in the first chunk in which the AA transition rate is high. To circumvent this issue, we tolerated some approximations in the jump detection by counting a hit when there was a true generative jump within a window of ±5 stimuli around the reported jump location, and a false alarm otherwise. This same window size was used throughout our data analysis, and other choices did not change the qualitative findings.

In line with the ROC approach, we computed, for each subject, the difference in hit rate minus false alarm rate, known as the informedness index. Informedness is bounded between -1 and 1, with values higher than 0 denoting a detection better than chance; and lower than 0 a detection worse than chance. A t-test on informedness revealed that the mean value was significantly larger than zero. However, to make sure that such a result was unlikely to emerge by chance from the detection characteristics of our subjects and the generative structure of our sequences, we adopted a more conservative permutation-based approach. We computed a null (chance-level) t-value distribution for informedness by keeping subject reports unchanged but randomly regenerating (10000 times) the stimulus sequence. The p-value reported in the text corresponds to the probability of observing a t-value equal or higher under the null distribution, indicating how likely it is that the result is due to chance.

We followed up the results of the ROC analysis by inspecting the posterior probability of jump estimated by the Ideal Observer in trials corresponding to the subjects' hits, misses, false alarms and correct rejections. More precisely, since we tolerated a margin of ±5 stimuli in the subjects' jump detection, we compared the subjects' report with the posterior probability that a jump occurred in a window of ±5 stimuli around each observation, see Fig 2E for an example session. For hits and false alarms, we took the posterior probability of a jump at the position reported by subjects, given the sequence they had observed when they reported it. It is less straightforward for misses and correct rejections since, precisely, jumps were never reported at these positions. We thus estimated for each subject the typical latencies of jump report and we averaged over this list of latencies to compute the posterior probability of jump at each position corresponding to a correct rejection or miss.

### Regression analyses

To assess the accuracy of the subjects' probability estimates and confidence ratings, we used several regressions against predictor variables. The significance of these regression analyses was estimated by computing regression coefficients at the subject-level as a summary statistic and then comparing these coefficients against zero with a two-tailed t-test at the group level (t and p-values are reported in the text). All regression models included a constant and the z-scored regressors of interest.

The multiple regressions corresponding to Fig 6 deserves more details. In Fig 6A the estimation revision is the absolute difference of the Ideal Observer probability estimates between two consecutive similar transitions. Consecutive transitions are not necessarily consecutive stimuli (e.g. the transition 'from A' in ABBBBAA). In Fig 6B, the jump-wise count of samples was also made per transition type. For this count, a log-scale was used since it is an analytical result that, on average, confidence (the Ideal Observer log-precision) should increase linearly with the log-number of samples. We based this count on the Ideal Observer. However, the Ideal Observer does not estimate a binary variable (there is a jump vs. there is not), instead it computes the continuous posterior probability that a jump occurs at each position of the observed sequence, and it revises this estimate each time a new observation is made. We therefore transformed the posterior probability estimates (a two-dimensional matrix, see Fig 2E for an example) into discrete jumps. The thresholded (two-dimensional) posterior probability serves to identify when the sequence should be interrupted to report a jump and what should be the location of the reported jump, e.g, report at trial W that a jump occurred at position Z (thus W-Z trials ago). The posterior jump probability being relatively smooth (e.g. in Fig 2E), the thresholding forms patches. Each of these patches corresponds to a jump; the reported W and Z corresponds to the coordinates of the upper limit of each patch. We used a Receiver Operating Characteristic to identify the threshold (posterior probability = 0.25) that maximized the accuracy of this discretization, with respect to the actual generative jumps: we searched the threshold that resulted in the maximal difference between hit and false alarm rates.

### Within-subject correlation between estimation and confidence accuracies

We took as an estimate of single-trial accuracy, the un-signed error (i.e. the distance) between the subject estimate and the Ideal Observer estimate. The probability estimates in both the subjects and the Ideal Observer are expressed on the same probability scale: they can be compared directly. This is not the case for confidence: the scale for the Ideal Observer is normative, it is the log-precision which can be potentially infinite; by contrast for subjects the scale was bounded and qualitative, the mapping between confidence levels and the scale is thus highly subjective. To express the Ideal Observer and the subject confidence on a common scale, we adjusted their offset and scaling based on a linear fit.

For each subject, the single-trial accuracies in probability estimates and confidence ratings were taken into a Pearson correlation over trials. The resulting correlation coefficients could then have been taken into a classical t-test; however, we wanted to estimate to what extent the correlation would be positive due to a systematic mapping between probability estimates and confidence ratings. We thus devised two permutation-based estimations, each corresponding to a null-hypothesis distribution of the correlation of accuracies between probability estimates and confidence ratings. Shuffling #1 (Fig 5C, middle) preserved the mapping but disrupted the sequence, by keeping pairs of probability estimates—confidence ratings and shuffling their order in the sequence separately for the Ideal Observer and the subjects. Shuffling #2 (Fig 5C, right) disrupted both the mapping and the sequence by shuffling the trials independently for probability estimates and confidence ratings, thus removing any correlation between them. 10000 distinct permutations were used to estimate each null distribution. Given that the shuffling was applied within-subject, we computed the null t-distribution for the paired differences between 'Observed data' and 'Shuffling keeping pairs'. The 'Full shuffling' resulted in values close to 0 for all participants so that the estimated null t-distribution was equivalent to the parametric t-distribution; tests against the 'Full shuffling' null were thus classical t-tests against 0. P-values in Fig 5C correspond to one-tailed t-test.

### Ideal Observer

We derived mathematically the optimal observation-driven estimates of the transition probabilities and jump locations: the so-called Ideal Observer. This optimal inference relies on Bayesian principles and returns a distribution of estimates *p*(*θ* | *y*), i.e. the posterior distribution of the transition probability, θ, at each time step in the experiment, given the observed sequence of stimuli, *y*. From this distribution, we derive the expected value of the inferred transition probability: *μ* = ∫ *θp*(*θ* | *y*)*dθ* and the confidence in that estimation, which we defined as its log-precision: -log(∫(*θ* − *μ*)^2^ *p*(*θ* | *y*)*dθ*).

We designed two algorithms for this Ideal Observer: a sampling approach and an iterative approach. The iterative approach was used to double check the sampling approach: both provided numerically similar values of probability estimates, confidence levels and jump location. The sampling approach explicitly computes the likelihood of possible decompositions of the sequence into chunks, whereas the iterative approach computes the likelihood that a jump occurred at any given position, independently from the other potential positions. The sampling approach is computationally slower but it allows a straightforward estimation of jump-related statistics used here: 1) The likelihood that a jump occurred *around* a given position, e.g. within a window of ±5 stimuli; 2) The variance of the estimated length of the current chunk, which reflects the precision of the knowledge of the observer about the last jump location. The derivation of each algorithm is presented in detail below. Computations were performed numerically in Matlab using regular grids.

### Sampling algorithm for the Ideal Observer

If we assume that the transition probabilities generating the sequence are stable over time, then the inference can be computed analytically: the posterior distribution is a function of the number of transitions observed in the sequence. The formula is derived in the first sub-section below. However, sequences in the task were generated with jumps. For a given partition, the inference of transition probabilities can be made chunk-wise using the above-mentioned formula. Such an inference is conditional in the sense that it is computed given a particular partition. However, the partition itself is unknown and must be inferred from the sequence observed. The estimation of the transition probabilities must therefore factor out the uncertainty in the partition, which is achieved by marginalizing the conditional inference over all partitions:

$$p\left(\theta |y_{1,}\ldots ,y_{t}\right)=\sum_{\pi }p\left(\theta |y_{1,}\ldots ,y_{t},\pi \right)p\left(\pi |y_{1,}\ldots ,y_{t}\right)$$ (1)

Where y is the sequence of A and B stimuli, θ = [θ_A|B_, θ_B|A_] are the transition probabilities 'from B to A' and 'from A to B' respectively, and π is a partition describing the location of jumps. The 1^st^ term of the sum is thus the conditional posterior distribution of transition probabilities given a particular partition of the sequence; the second term is the posterior probability of this partition.

The sequence length being 380, there are 2^380^ possible partitions of the data. The exact inference would require that we compute the sum over these 2^380^ partitions. It is computationally intractable and actually not necessary: most partitions are very unlikely and contribute little to the sum. The posterior distribution of transition probabilities can thus be approximated numerically by averaging the conditional posterior distributions of transition probabilities over a subset of partitions sampled uniformly [22]. The second subsection below shows how to sample uniformly from the posterior distribution of partitions.

#### Posterior inference of transition probabilities within a chunk

The posterior probability of θ can be computed with Bayes rule:

$$p\left(\theta |y\right)=\frac{p\left(y|\theta \right)p\left(\theta \right)}{p\left(y\right)}$$ (2)

Since p(y) is a scaling factor independent from the parameters to infer (θ), we only need to compute the likelihood and the prior. By design of the sequences, the likelihood of a stimulus in a particular chunk depends only on the transition probabilities and the identity of the previous stimulus. The likelihood of a sequence of stimuli can thus be written as the product of the likelihood of every transition in the sequence. As a result, the likelihood of a sequence, given the transition probabilities between stimuli, is entirely determined by the likelihood of the first stimulus and the number of transitions:

$$\begin{array}{l} p\left(y|\theta \right)=p\left(y_{1,}\ldots ,y_{n}|\theta \right)\\ \;=p\left(y_{1}|\theta \right)p\left(y_{2}|y_{1,}\theta \right)p\left(y_{3}|y_{2,}\theta \right)\ldots p\left(y_{n}|y_{n-1},\theta \right)\\ \;=p\left(y_{1}|\theta \right)\left[\theta_{A|B}^{N_{A|B}}{\left(1-\theta_{A|B}\right)}^{N_{B|B}}\right]\left[\theta_{B|A}^{N_{B|A}}{\left(1-\theta_{B|A}\right)}^{N_{A|A}}\right] \end{array}$$ (3)

The two terms outlined by square brackets correspond to beta distributions, their shape is controlled by the number of transition types (denoted N_x|y_). To keep the benefit of having beta distributions in our formula, we used a conjugate prior distribution. The parameters of the (beta) prior distribution can be interpreted as prior transition counts. The likelihood of the first stimulus can be considered as independent from the transition probabilities (as if it belonged to the previous chunk), so that the posterior is actually proportional to the product of two beta distributions:

$$\begin{array}{l} p\left(y|\theta \right)p\left(\theta \right)\propto \theta_{A|B}^{N_{A|B}+N_{A|B}^{*}}{\left(1-\theta_{A|B}\right)}^{N_{B|B}+N_{B|B}^{*}}\;\theta_{B|A}^{N_{B|A}+N_{B|A}^{*}}{\left(1-\theta_{B|A}\right)}^{N_{A|A}+N_{A|A}^{*}}\\ \;\propto Beta\left(\theta_{A|B}|N_{A|B}+N_{A|B}^{*}+1,N_{B|B}+N_{B|B}^{*}+1\right)Beta\left(\theta_{B|A}|N_{B|A}+N_{B|A}^{*}+1,N_{A|A}+N_{A|A}^{*}+1\right) \end{array}$$ (4)

Where N*_x|y_ denotes the prior transition count. In practice, we used a non-informative prior: every transition probability is considered with the same plausibility a priori (all N* are zeros, so that the prior distribution is a flat beta distribution).

#### Sampling from the posterior distribution of partitions

Conditionally on jumps, the sequence of stimuli within each chunk is independent from the other chunks, so that the likelihood of a sequence given some jumps is the product of the likelihood of each chunk defined by these jumps:

$$\begin{array}{l} p\left(y|\pi \right)=\prod_{k=1}^{N_{c}}p\left(y_{i\in \pi \left(k\right)}\right)\\ \;=\prod_{k=1}^{N_{c}}ML\left(\pi \left(k\right)\right) \end{array}$$ (5)

Where π(k) denotes the list of indices of the stimuli belonging to the k-th chunk. We introduce the notation ML(π(k)) to denote the marginal likelihood of the sequence in the k-th chunk, i.e. the likelihood of this sequence after marginalizing over the range of transition probabilities (this integral has an analytical solution, involving gamma functions).

Eq (5) shows that the likelihood of a sequence given a partition is the product of the marginal likelihoods of the chunks of this partition. Say that we want to introduce a new jump in this partition, at position i. To compute the likelihood of this new partition, which differs from the previous one by only one new jump, we simply have to identify the chunk corresponding the i-th position and to replace in (5) the marginal likelihood of this chunk (denoted c below) by the product of marginal likelihoods of the two chunks defined by the new jump (denoted c_1_ and c_2_ below). We can thus compute the relative posterior probability that a jump occurred at position i (denoted J_i_ = 1, which occurs with prior probability p_J_) conditionally on the jumps at the other positions (denoted J_-i_):

$$\begin{array}{l} p\left(J_{i}|J_{-i},y\right)\propto p\left(y|J_{i},J_{-i}\right)p\left(J_{i}\right)\\ p\left(J_{i}|J_{-i},y\right)\propto ML\left(c_{1}\right)ML\left(c_{2}\right)p_{J}\;if\;J_{i}=1\\ \;\propto ML\left(c\right)\left(1-p_{J}\right)\;if\;J_{i}=0 \end{array}$$ (6)

For a given partition, we can quantify with Eq (6) whether adding or deleting a jump at a given position would increase the likelihood of the partition. Based on this property, we designed an algorithm which iteratively improves the sampling from the posterior J: a Gibbs sampler [22]. We initialized the Gibbs sampler without jump. Convergence was achieved within few iterations, reflecting that there are only few positions in the sequence around which jumps are likely. Indeed, few jumps were used to generate the sequences. Using only 200 iterations was enough to sample accurately from the posterior distribution (we discarded the first 20 iterations as a warm-up).

### Iterative algorithm for the Ideal Observer

It is not necessary to decompose the sequence explicitly into a partition to compute the posterior θ distribution given the stimuli observed. Indeed, if we know θ at position t in the sequence, then at position t+1, θ should remain the same if no jump occurred, or be different if a jump occurred. In case a jump occurred, the new θ is sampled from the prior distribution and the likelihood can be assessed given the (t+1)-th stimulus. In that case, the observations made *before* t become no longer needed to estimate θ *after* t. This so-called Markov property makes it possible to estimate θ iteratively, by going *forwar*d: at stimulus t+1, we update the estimate made at time t, based on the new observation.

In the following we derive the *forward* algorithm to estimate θ, the transition probabilities. We also derive a *backward* algorithm to estimate the likelihood of jumps in the observed sequence. Note that both algorithms are provided with the exact same observations as those presented to the subject. In particular, the backward sweep does not benefit from extra stimuli not yet observed by the subject, but simply processes the information received by moving backward in time.

#### Forward algorithm to estimate the transition probabilities

We note θ_t_ = {θ_t, 1|0_, θ_t, 0|1_} the estimate of the two transition probabilities made at time t. The estimation problem can be recast as a Hidden Markov Model by the discretization of θ into some so-called states: the continuous {θ_t, 1|0_, θ_t, 0|1_} are discretized into some states {x^1^_t_, x^2^_t_}, where subscripts denote the t-th position (or stimulus) of the sequence and supscripts 1 and 2 denote the two transition probabilities.

The *forward* algorithm updates iteratively the joint probability, denoted α, of the states and the stimuli observed so far (noted y_1:t_):

$$\alpha_{t}\left(x_{t}^{1},x_{t}^{2}\right)=p\left(x_{t}^{1},x_{t}^{2},y_{1:t}\right)$$ (7)

The iterative property can be seen by marginalizing over past states, in particular when the matrix notation is used:

$$\begin{array}{c} \begin{array}{l} \alpha_{t}\left(x_{t}^{1},x_{t}^{2}\right)=\sum_{x_{t-1}^{1},x_{t-1}^{2}}p\left(x_{t-1}^{1},x_{t-1}^{2},x_{t}^{1},x_{t}^{2},y_{1:t}\right)\\ \;=\sum_{x_{t-1}^{1},x_{t-1}^{2}}p\left(x_{t-1}^{1},x_{t-1}^{2},y_{1:t-1}\right)p\left(x_{t}^{1},x_{t}^{2}|x_{t-1}^{1},x_{t-1}^{2}\right)p\left(y_{t}|x_{t}^{1},x_{t}^{2},y_{t-1}\right)\\ \;\alpha_{t}=L\cdot \left(T^{T}\alpha_{t-1}\right) \end{array} \end{array}$$ (8)

In the second line of (8), the first probability is α_t-1_, the second term is the probability of the transition between states and the last term is the likelihood of the current observation y_t_ given some transition probabilities {x^1^_t_, x^2^_t_} and the previous observation y_t-1_. We achieve a more compact formula with the matrix notion (bold font), where the dot denotes the dot product, **L** is the column vector of likelihood and **T** is the matrix of transition probability between states. A diagonal element in **T** corresponds to the probability of staying in the same state, when no jump occurred (p_J_).

#### Estimation of jump positions with a backward algorithm

In Eq (8), the terms in the sum can be grouped, depending on whether they reflect the occurrence of a jump or not: the terms corresponding to the absence of a jump at position t come from the diagonal elements of **T**, the other come from the off-diagonal elements of **T**. The joint probability α_t_ can thus be re-written to tease apart the occurrence and absence of a jump at position t:

$$\alpha_{t}\left(x_{t}^{1},x_{t}^{2}\right)=\alpha_{t}\left(x_{t}^{1},x_{t}^{2},J_{t}=0\right)+\alpha_{t}\left(x_{t}^{1},x_{t}^{2},J_{t}=1\right)$$ (9)

However, the observations y_1:t_ convey little information on whether a jump occurred at t: by definition of a change, the observations made *after* position t are more informative. The *forward* algorithm at position t does not consider observations made *after* t; however the *backward* algorithm does. We thus use it to complement the *forward* algorithm and to estimate the likelihood of jumps.

The *backward* algorithm updates iteratively the likelihood of observing future observations (noted y_t+1:N_) given the current state {x^1^_t_, x^2^_t_}, which we note β:

$$\beta_{t}\left(x_{t}^{1},x_{t}^{2}\right)=p\left(y_{t+1:N}|x_{t}^{1},x_{t}^{2}\right)$$ (10)

The iterative property can be seen by marginalizing over future states, and again, using the matrix notation:

$$\begin{array}{l} \beta_{t-1}\left(x_{t-1}^{1},x_{t-1}^{2}\right)=\sum_{x_{t}^{1},x_{t}^{2}}p\left(y_{t:N},x_{t}^{1},x_{t}^{2}|x_{t-1}^{1},x_{t-1}^{2}\right)\\ \;=\sum_{x_{t}^{1},x_{t}^{2}}p\left(x_{t}^{1},x_{t}^{2}|x_{t-1}^{1},x_{t-1}^{2}\right)p\left(y_{t+1:N}|x_{t}^{1},x_{t}^{2}\right)p\left(y_{t}|x_{t}^{1},x_{t}^{2},y_{t+1}\right)\\ \;\beta_{t}=T\left(L\cdot \beta_{t}\right) \end{array}$$ (11)

In this sum, we can identify the probability of the transitions between states (T), β_t_, and a likelihood term (L). The likelihood term here is not as straightforward as in Eq (8), but it can be decomposed as follows:

$$p\left(y_{t}|x_{t}^{1},x_{t}^{2},y_{t+1}\right)=\frac{p\left(y_{t+1}|x_{t}^{1},x_{t}^{2},y_{t}\right)p\left(y_{t}|x_{t}^{1},x_{t}^{2}\right)}{p_{J}p\left(y_{t+1}|y_{t}\right)+\left(1-p_{J}\right)p\left(y_{t+1}|y_{t},x_{t+1}^{1}=x_{t}^{1},x_{t+1}^{2}=x_{t}^{2}\right)}$$ (12)

As for the *forward* algorithm, we can rearrange the sum in Eq (11) to tease apart the occurrence and absence of a jump:

$$\begin{array}{l} \beta \left(x_{t}^{1},x_{t}^{2}\right)=p\left(y_{t+1:N},J_{t}=0|x_{t}^{1},x_{t}^{2}\right)+p\left(y_{t+1:N},J_{t}=1|x_{t}^{1},x_{t}^{2}\right)\\ \;=\beta \left(x_{t}^{1},x_{t}^{2},J_{t}=0\right)+\beta \left(x_{t}^{1},x_{t}^{2},J_{t}=1\right) \end{array}$$ (13)

The *forward* and *backward* quantities can be combined to estimate the posterior distribution of the transition probabilities and the probability of a jump, at position k based on observations y_1:t_ (with k≤t):

$$\begin{array}{l} \gamma_{t}\left(x_{k}^{1},x_{k}^{2},J_{k}\right)=p\left(x_{k}^{1},x_{k}^{2},J_{k}|y_{1:t}\right)\\ \;=p\left(x_{k}^{1},x_{k}^{2},J_{k}|y_{1:k}\right)p\left(y_{k+1:t}|x_{k}^{1},x_{k}^{2},J_{k}\right)\\ \;=p\left(x_{k}^{1},x_{k}^{2},J_{k}|y_{1:k}\right)\frac{p\left(y_{k+1:t},J_{k}|x_{k}^{1},x_{k}^{2}\right)}{p\left(J_{k}\right)}\\ \;=\frac{\alpha \left(x_{k}^{1},x_{k}^{2},J_{k}\right)}{\sum_{x_{k}^{1},x_{k}^{2},J_{k}}\alpha \left(x_{k}^{1},x_{k}^{2},J_{k}\right)}\frac{\beta \left(x_{k}^{1},x_{k}^{2},J_{k}\right)}{p\left(J_{k}\right)} \end{array}$$ (14)

Note p(J_k_) = p_J_ when J_k_ = 1 and (1-p_J_) otherwise. We can obtain the posterior probability of J_k_, irrespective of the transition probabilities {x^1^_t_, x^2^_t_} by marginalizing γ over {x^1^_t_, x^2^_t_}.

## Supporting Information

S1 Text Description of the data file provided as Supporting Information. (PDF)

S1 Dataset Data file. (MAT)
