# Supplementary material for: The Sense of Confidence during Probabilistic Learning: A Normative Account
Source: PLoS Comput Biol. 2015 Jun 15;11(6):e1004305. doi: 10.1371/journal.pcbi.1004305 (PMC4468157; doi:10.1371/journal.pcbi.1004305)
Supplement: S1 Text — (PDF) [file pcbi.1004305.s001.pdf]

The file DataSetAllSubjects.mat is in the MATLAB 7.3 format. It contains a structure labelled 'data' of size 18x4 (including our 18 subjects, with 4 blocks each). Each cell contains several fields: 'sequence': the binary sequence of stimuli (coded as 1s and 2s); 'generative_p1g2' and 'generative_p2g1': the generative transition probabilities p(1|2) and p(2|1) used to generate the sequence of stimuli; 'modality': the block type (either visual or audio); 'SubEstimate_p1': the subjective estimate of p(1) (answer to the 1st question, see Fig. 1); 'SubConfidence': the subjective confidence level (answer to the 2nd question, see Fig.1; the scale was coded such that 0 is the minimum and 1 the maximum); 'JumpReport': the subject's report that a jump occurred (indicating the number of stimuli observed since the jump putatively occurred, at the moment of the report).
